# Associations between Relationship Quality and Mental Health during COVID-19 in the United Kingdom

**DOI:** 10.3390/ijerph18062869

**Published:** 2021-03-11

**Authors:** Christoph Pieh, Thomas Probst, Sanja Budimir, Elke Humer

**Affiliations:** 1 Department for Psychotherapy and Biopsychosocial Health, Danube University Krems, 3500 Krems an der Donau, Austria; thomas.probst@donau-uni.ac.at (T.P.); sanja.budimir@donau-uni.ac.at (S.B.); elke.humer@donau-uni.ac.at (E.H.); 2 Department of Work, Organization and Society, Ghent University, 9000 Gent, Belgium

**Keywords:** relationship quality, COVID-19, mental health, life quality, well-being

## Abstract

This study investigated the association of relationship quality with several well-being measures during the COVID-19 lockdown in the United Kingdom. An online survey was conducted on a study sample (*n* = 682) measuring relationship quality with the Quality of Marriage Index, and well-being measures including quality of life (WHO-QoL BREF), well-being (WHO-5), perceived stress (PSS-10), depressive (PHQ-9), and anxiety (GAD-7) symptoms. Good relationship quality goes along with higher scores in well-being (WHO-5), quality of life (WHO-QoL BREF), psychological domain, physical health, social relationships, environment, and reduced scores in stress (PSS-10), depressive (PHQ-9) and anxiety (GAD-7) symptoms compared with poor relationship quality (*p* < 0.001). Moreover, 21% of participants living in relationships with poor quality stated that they drink significantly more alcohol since the initial COVID-19 restrictions, compared to 10% of participants with good quality (*p* = 0.002). Living in a good relationship seems to be an advantage, whereas those with poor relationship quality are particularly more burdened and drink significantly more alcohol during the COVID-19 lockdown.

## 1. Introduction

The lockdown due to the COVID-19 pandemic limited and focused social interactions to interactions within a household, resulting in increased frequency of contact between partners. We aimed to evaluate the effect of relationship quality on various mental health and well-being indicators during the COVID-19 lockdown.

Relationship status and relationship quality have been reliably associated with health benefits and mental well-being [1,2,3]. For example, marital quality significantly predicts well-being measures, such as satisfaction with life, stress, or depression, but also physiological parameters, such as blood pressure [4]. Vice versa, loneliness can lead to various psychiatric disorders, such as depression or sleep problems, as well as physical disorders, such as diabetes or cardiovascular diseases [5].

The current coronavirus disease 2019 (COVID-19) pandemic and the governmental restrictions constitute an extraordinary situation for relationships and mental well-being [6]. To prevent the uncontrolled spreading of the virus, most governments imposed a national lockdown. In the UK, the lockdown became obligatory on 24 March 2020. During the lockdown, the population was required to adhere to quarantine with only a few exceptions justifying leaving the house (e.g., shopping for food and other necessities; exercising alone or with someone from the same household; for medical reasons including providing care to others; and traveling to and from work).

As known as a result of previous disasters, such challenging times can have an impact on relationships, marriage, and divorce rates [7]. However, as the COVID-19 lockdown is unique and difficult to compare with, the impact of relationship quality on well-being is unclear. However, it is likely that during lockdown measures the relationship quality has a particular influence on well-being. Therefore, the current study evaluates the impact of relationship quality on well-being indicators during the COVID-19 lockdown in the UK.

## 2. Materials and Methods

A representative study sample according to age, gender, education, and region for the UK was recruited through the Qualtrics^®^ population survey platform by quota sampling on 1006 participants. From the total sample, those living in a relationship were included in this study. Relationship quality was assessed during the lockdown (i.e., 4 weeks after its start, 21 April–31 April) with the Quality of Marriage Index (QMI) [8] using the recommended cut-off score of ≥34 to distinguish between good and poor relationship quality [9]. Furthermore, well-being (WHO Well-being Index: WHO-5) [10], quality of life (WHO-QoL BREF) [11], stress (Perceived Stress Scale: PSS-10) [12], depressive symptoms (Patient Health Questionnaire: PHQ-9) [13], and anxiety (Generalized Anxiety Disorder 7 scale: GAD-7) [14] were measured. Additionally, the drinking behavior of alcoholic beverages was assessed using the following question: “Has your drinking behavior of alcoholic beverages such as beer, wine, or spirits changed since the initial restrictions?”. Participants had to choose between four answer options: “I don`t drink alcohol, I drink as much alcohol as I did before”, “I drink significantly less alcohol than before”, “I drink significantly more alcohol than before”. All data were analyzed using SPSS^®^ version 26 (IBM Corp, Armonk, NY, USA). Descriptive statistic was used to describe the demographic characteristics and scales mean values. Mental health scale data were analyzed for normality using the Shapiro–Wilk test, which confirmed non-normal distributions. As for large sample sizes, non-parametric tests are not recommended [15], differences between good and poor relationships concerning all metric outcome variables were analyzed using t-tests, whereas chi-squared tests were applied for nominal data. The two-sided significance level was set at <0.05. Cohen’s d was calculated for the effect size.

## 3. Results

Within the study sample (*N* = 1006), *n* = 682 are currently living in a relationship comprising 52.8% females and 47.2% males. Further sample characteristics are summarized in Table 1.

According to the QMI, *n* = 500 are living in a relationship with good and *n* = 182 are living in a relationship with poor quality. Chi-squared tests revealed no differences concerning age, gender, educational level, job situation, and net income between participants living in good or poor relationships (all *p* values ≥ 0.09). T-tests revealed that individuals with good relationships had significantly better outcomes in all measured scales than those with a poor relationship (all *p* values < 0.001; effect sizes ranged from *d* = 0.37 to *d* = 1.21; Table 2).

Additionally, it was found that 21% of participants who are living in a relationship with poor quality stated that they now drink significantly more alcohol than before the lockdown measures, compared to 10% of participants with good relationship quality (*χ*^2^(3) = 14.72; *p* = 0.002; Table 3).

## 4. Discussion

The current study explored the effect of the relationship quality on well-being indicators during the COVID-19 lockdown in the UK. Results suggest that good relationship quality is positively associated with well-being and mental health during the challenging situation around the COVID-19 pandemic. Individuals living in a relationship with a poor quality experience more of the mental burden during the COVID-19 lockdown and drink more alcohol during the COVID-19 lockdown.

The observed association of good relationship quality and mental well-being corresponds to previous research [5]. Previous studies revealed associations between relationship quality and mental well-being, with better mental health in married individuals compared to never-married ones [2]. It has also been reported that married women and men consume less alcohol and have fewer drinking problems compared to singles or divorced individuals [16]. However, it seems that quality of the relationship, rather than being married per se, is crucial for well-being, as unhappily married individuals have been shown to have worse mental health than singles [1,4]. Our findings indicate that the beneficial association of sharing life with a partner and mental well-being is present only for those living in a relationship with good quality, whereas those living in a relationship with a poor quality showed deteriorated mental health, well-being, and life quality.

A main limitation of the study is that no causal conclusions are possible about changes in the effect of relationship quality on well-being and mental health due to the COVID-19 restrictions. A longitudinal study with a further survey conducted before the COVID-19 lockdown would have been more appropriate to draw causal conclusions whether relationship quality affected well-being or whether well-being affected relationship quality or both. A further limitation is that only self-rating scales and no clinician-based assessments were applied. Another limitation is the unequal sample size of participants among the relationship quality groups (*n* = 182 in the group with poor relationships vs. *n* = 500 in the group with good relationships), which impedes the robustness of statistical tests.

## 5. Conclusions

During a COVID-19 lockdown, relationship quality seems to be positively associated with well-being and mental health. Individuals with poor relationship quality are not only particularly more burdened compared with those with good relationship quality, but also drink significantly more alcohol during the COVID-19 lockdown.

## Figures and Tables

**Table 1:** Study sample characteristics (*n* = 682).

Variable	*N*	%
Gender		
Women	360	52.8
Men	322	47.2
Age		
18–24	47	6.9
25–34	147	21.6
35–44	135	19.8
45–54	131	19.2
55–64	120	17.6
65+	102	15.0
Marital Status		
Single	68	10.0
Separated	2	0.3
Married	405	59.4
Divorced	18	2.6
Living as married	187	27.4
Widowed	2	0.3
Region		
North East	33	4.8
North West	84	12.3
Yorkshire and The Humber	63	9.2
East Midlands	56	8.2
West Midlands	58	8.5
East of England	61	8.9
London	66	9.7
South East	92	13.5
South West	58	8.5
Wales	36	5.3
Scotland	58	8.5
Northern Ireland	17	2.5
Education		
None at all	9	1.3
Elementary school	23	3.4
Trade/technical/vocational training	107	15.7
High school or equivalent	267	39.1
College	81	11.9
Bachelor’s degree	109	16.0
Master’s degree	66	9.7
Doctoral degree	12	1.8
Professional degree (MD, JD, etc.)	8	1.2
Net household income		
less than GBP 900,-	60	8.8
from GBP 900,- equal to GBP 1800,-	209	30.6
more than GBP 1800,- equal to GBP 2700,-	202	29.6
more than GBP 2700,- equal to GBP 3600,-	115	16.9
more than GBP 3600,-	96	14.1
Job situation		
No paid work (also before the lockdown)	140	20.5
No paid work since the lockdown	161	23.6
Home office	132	19.4
Paid work at the workplace (no home office)	100	14.7
Paid work, but reduced hours since lockdown	48	7.0
Retired	101	14.8

**Table 2:** Measures of well-being and mental health during the COVID-19 lockdown in individuals with good (*n* = 500) or poor (*n* = 182) relationship.

Variable	Unit	Good Relationship	Poor Relationship	Statistic
WHO-5	M	14.46	10.79	*t*(680) = 7.60;
	SD	5.61	5.47	*p* < 0.001; *d* = −0.66
WHO-QoL BREF				
Psychological	M	65.66	49.47	*t*(680) = 9.97;
	SD	18.74	18.77	*p* < 0.001; *d* = −0.86
Physical health	M	69.11	61.58	*t*(680) = 4.33;
	SD	20.52	18.98	*p* < 0.001; *d* = −0.37
Social relationships	M	72.40	48.63	*t*(284.9) = 13.06;
	SD	18.81	21.79	*p* < 0.001; *d* = −1.21
Environment	M	69.53	56.28	*t*(680) = 8.97;
	SD	17.17	16.71	*p* < 0.001; *d* = −0.78
PSS-10	M	15.93	20.83	*t*(365.4) = −8.10;
	SD	7.69	6.71	*p* < 0.001; *d* = 0.66
PHQ-9	M	7.26	12.15	*t*(680) = −7.88;
	SD	7.04	7.55	*p* < 0.001; *d* = 0.68
GAD-7	M	6.81	10.70	*t*(680) = −7.20;
	SD	6.13	6.50	*p* < 0.001; *d* = 0.62

*p*: *p*-values (two-tailed); M: mean score; SD: standard deviation, *t*: *t*-test; *d*: Cohen’s d, an effect size, GAD-7: Generalized Anxiety Disorder 7 scale; PHQ-9: Patient Health Questionnaire 9 scale; PSS-10: Perceived Stress Scale 10; WHO-5: Well-being questionnaire of the World Health Organization (WHO); WHO-QoL BREF: Quality of Life questionnaire of the World Health Organization (WHO).

**Table 3:** Drinking behavior of alcoholic beverages since the initial COVID-19 restrictions in individuals with good (*n* = 500) or poor (*n* = 182) relationship.

Alcohol Consumption	Unit	Good Relationship	Poor Relationship	Statistic
I don’t drink alcohol	F	156	50	*χ*^2^(3) = 14.72; *p* = 0.002
	%	31.2	27.5	
I drink as much alcohol	F	235	75	
as I did before	%	47.0	41.2	
I drink significantly less	F	60	19	
alcohol than before	%	12.0	10.4	
I drink significantly more	F	49	38	
alcohol than before	%	9.8	20.9	

*p*: *p*-values (two-tailed); F: frequencies; %: percentages; *χ*^2^: Chi-squared-test.

## Data Availability

The raw data supporting the conclusion of this article will be made available by the authors upon reasonable request.
